# Not All in Vein: Oral Antibiotics for Diabetic Foot Osteomyelitis: A Narrative Review

**DOI:** 10.3390/jcm14051405

**Published:** 2025-02-20

**Authors:** Benoit Gachet, Marcos C. Schechter, David G. Armstrong, Olivier Robineau, Eric Senneville

**Affiliations:** 1 Infectious Diseases Unit, Gustave Dron Hospital, F-59200 Tourcoing, France; bgachet@ch-tourcoing.fr (B.G.);; 2 French National Referent Centre for Complex Bone and Joint Infections, CRIOAC Lille-Tourcoing, F-59000 Lille, France; 3 EA2694, Lille University, F-59000 Lille, France; 4 Grady Memorial Hospital, Atlanta, GA 30303, USA; 5 Department of Medicine, Division of Infectious Diseases, Emory University, Atlanta, GA 30322, USA; 6 Department of Surgery, Keck School of Medicine, University of Southern California, Los Angeles, CA 90007, USA; armstrong@usa.net

**Keywords:** diabetic foot osteomyelitis, oral antibiotics, intravenous antibiotics, diabetic foot infections, antimicrobial therapy

## Abstract

Introduction: Osteomyelitis is a severe complication of diabetes-related foot ulcers (DFUs) often managed with antibiotic therapy and surgical resection of the infected bone. Areas of research: While intravenous (IV) antibiotics have been the traditional approach for bone and joint infections in general, randomized clinical trials have shown that, overall, oral antibiotics are non-inferior to IV antibiotics. While comparisons between oral antibiotics are generally lacking, the data suggest that oral antibiotics with high bioavailability and bone penetration ratios should be prioritized for osteomyelitis treatment, including diabetic foot osteomyelitis (DFO). Oral regimens reduce hospital stays, avert catheter-related complications, and decrease treatment costs while improving patient satisfaction and quality of life. Despite these advantages, IV antibiotics remain widely used, partly due to clinical tradition and concerns about oral absorption in individuals with diabetes. Current guidelines recommend transitioning to oral therapy once systemic signs improve, but robust data supporting oral-only regimens for DFO treated non-surgically remain limited. Conclusions: Oral antibiotics represent a safe and effective alternative to IV therapy for many patients with DFO, particularly when high-bioavailability agents are used. Further well-designed studies are needed to validate their efficacy in non-surgical DFO management and inform clinical guidelines.

## 1. Introduction

Osteomyelitis is associated with diabetes-related foot ulcer (DFUs) in 10% to 68% of cases according to the infection severity [1]. Osteomyelitis of the foot in people with diabetes (DFO) has long been considered as a catastrophic situation that still justifies prompt surgical intervention including amputation and a prolonged intravenous antibiotics course [2,3]. During the last decades, practice has changed and non-surgical treatment or conservative surgery (i.e., purposively not resecting all infected bones to preserve the foot biomechanics) and oral antibiotics have been progressively adopted [4]. Data from recent studies suggest that both amputation and intravenous antibiotic therapy may not be necessary as often as it was thought earlier and may not be as safe either. The better interactions between surgeons, pathologists, infectious diseases physicians, and microbiologists within multidisciplinary teams may explain this new trend [5].

Numerous randomized controlled trials (RCTs) have established that a brief intravenous course followed by oral therapy is as effective as intravenous-only therapy for osteomyelitis in general [6,7,8,9,10,11,12,13,14,15]. The fact is bacteria have no mechanisms, molecularly or physiologically, to “know” what route was used to administer antibiotics to a patient. They only “know” if enough antibiotic arrives at the site of infection to inhibit their growth. Bacteria lack the ability to refuse to stop growing merely because the antibiotic arrived at the site of infection after passing through the gastrointestinal tract rather than being injected directly into the vein. Thus, there is no biological plausibility to the superiority of intravenous-only therapy for DFO if it can be established that oral therapy results in adequate concentrations of antibiotics in bone. This lack of biological plausibility of intravenous-only superiority, combined with extensive and unanimous randomized controlled trial data, leaves little doubt about the efficacy of oral transitional therapy for chronic osteomyelitis. Switching from intravenous to oral antibiotics, or using oral-only regimens, can reduce catheter-associated bloodstream infections and thrombophlebitis. It can also shorten hospital stays, lower nursing care costs, ease the pharmacy workload, and enhance patient satisfaction and quality of life [16,17]. Among clinical studies that have established similar outcomes in patients treated for chronic osteomyelitis with oral versus intravenous antibiotic therapy, the OVIVA study is the largest RCT of oral sequential therapy for osteomyelitis [13]. OVIVA not only established the non-inferiority of oral therapy with respect to clinical cure, but oral therapy resulted in markedly fewer adverse events and cost. From the perspective of the patient, therefore, when doctors insist on intravenous-only therapy for DFO, it is like they are self-administering a nocebo for their own anxiety, at the expense of increasing anxiety for their patients [18].

The present narrative document aims at reviewing the current data regarding oral versus intravenous antibiotics for the treatment of people with bacterial osteomyelitis and examining whether they can apply to people with DFO, including those treated with conservative surgery or non-surgically.

### 1.1. Do Intravenous and Oral Administration of Antibiotics Perform Equally in Terms of Bone Diffusion?

There are large data gaps in this area. While there are limited data regarding the associations between antibiotic bone penetration and treatment outcomes, it is intuitive that sufficient local antibiotic concentrations are important. It is important to realize that not all bones are created equal, and that antibiotic penetration into the cervical spine, calcaneus, and metatarsals may differ. Moreover, the diabetic foot presents a unique challenge due to the high co-prevalence of microvascular dysfunction [3].

The antibacterial activity of an antibiotic in bone tissues depends on multiple parameters. The bone concentration/minimal inhibitory concentration (MIC) ratio and the time during which this ratio is >1 between two administrations best predict the antibacterial activity for concentration-dependent antibiotics such as fluoroquinolones and aminoglycosides and time-dependent antibiotics such as beta-lactams and macrolides (depending on the dose and sensitivity of the bacteria, macrolides can become bactericidal) [19]. Another important parameter is the antibiotic bone/plasma concentration ratio (BPR) [19]. Since no barrier exists between blood and bone tissues, the amount of antibiotic that diffuses into bone tissues is driven by both BPR and the concentration reached into the plasma. BPR is influenced by factors such as the inflammatory status of the bone (i.e., infected versus infected) and the bone component (i.e., cancellous versus cortical), with the highest BPR values in inflamed cancellous bones [19]. Of note, the unique variable physicians can modify in case of systemic administration is the blood concentration of the antibiotic as BPR is a relatively fixed value. BPR values for most antibiotics have been reported by Landersdorfer et al. and updated by Thabit et al. in 2019 [19,20]. According to these authors, antibiotics with an average BPR > 0.3 include rifampicin, clindamycin, fluoroquinolones, tetracyclines, and linezolid, while values for glycopeptides, aminoglycosides, and beta-lactams range from 0.1 to 0.3.

The diffusion of antibiotics into bone tissues involved in DFO has been assessed for only a limited number of molecules, all administered intravenously. The concentration achieved in plasma and metatarsal cancellous bone of people with diabetes by linezolid administered twice daily at a dosage of 600 mg was assessed in steady-state conditions by a microdialysis technique [16]. Mean peak concentrations were, respectively, 16.6 ± 3.0 and 15.1 ± 4.1 mg/L. The ratio of the area under the concentration-time curve (AUC) of free linezolid from 0–12 h (fAUC_0–12_) in bone to the fAUC_0–12_ in plasma was 1.09 ± 0.11 [21]. The same research team used similar techniques to determine the concentrations of fosfomycin in metatarsal bone in nine patients scheduled for partial bone resection. Following a single dose of approximately 100 mg of fosfomycin per kg of body weight, the mean *C*_max_, *T*_max_, and AUC_0–6_ for bone and BPR were 96.4 mg/L, 3.9 h, 330.0 mg·h/L, and 0.43 ± 0.04, respectively [22]. Likewise, following daptomycin administration for at least four consecutive days at 6 mg/kg body weight in nine patients with DFO, the mean fAUC_0–16_ bone tissue/fAUC_0–16_ plasma ratio was 1.08 [23].

The best way to overcome BPR issues is to obtain high plasma concentrations by increasing antibiotic dosage, which is generally easier to do with IV antibiotics. This is, however, not always possible in cases of dose-related toxicity issues, as is reported for both aminoglycosides and vancomycin. By contrast, BPRs of beta-lactams do not exceed 20% [15], but their overall tolerance profile is good, which permits the use of high daily dosages. However, this only concerns parenteral beta-lactams since most oral beta-lactam agents exhibit low (i.e., < 80%) oral availability, which makes them unlikely to achieve adequate bone levels (Table 1) [24]. In their landmark paper based on a review of the studies in the field of chronic osteomyelitis published until 2012, Spellberg and Lipsky reported the values of bone penetration and BPR for oral and parenteral antibiotics established in clinical studies [25]. Overall, they showed that oral antibiotics with high bioavailability can attain bone concentration levels that exceed MICs of targeted organisms [25]. Another reason for selecting antibiotics with high oral bioavailability is to reduce the pressure on the gut flora. Indeed, antibiotics with incomplete bioavailability are more likely to exert prolonged antimicrobial pressure on the gut flora, including in the environment for molecules with high stability such as fluoroquinolones (FQ) and tetracyclines. The parenteral route of administration of FQ and tetracyclines does not, however, totally preclude this phenomenon because of their high biliary excretion [26,27].

### 1.2. What Are the Specificities of DFO That May Affect the Choice and Mode of Administration of Antibiotics in Patients with DFO?

Reduced oral absorption of antibiotics in people with diabetes, drug interactions, and low-compliance-related to tolerance issues, have been reported in patients treated for DFO [28,29,30]. The oral absorption of antibiotics among people with diabetes has been studied in people with tuberculosis, and studies suggest poor glycemic control may decrease the exposure of several antibiotics, including rifamycins and some fluoroquinolones [31,32]. This may be explained by the negative impact of hyperglycemia on intestinal motility and duodenal efflux pump P-glycoprotein combined with a delayed gastric emptying time and finally an increased mean absorption time [33]. The consequences vary from one antibiotic to another: while a decrease of ampicillin plasma concentrations has been reported, the oral bioavailability and the AUC of ciprofloxacin are unchanged and a prolonged *T*_max_, but no significant differences for *C*_max_ and AUC_0–24_ have been reported for rifampicin [33,34,35].

One may think that the frequent absence of pain in cases of diabetes-related foot infections (DFIs) is likely to reduce patients’ motivation and adherence to the antibiotic therapy. However, in a recent study, the adherence to antibiotic therapy assessed by an eight-item structured, self-reported medication adherence scale in people with diabetes was good, and independent of the type of infection (i.e., soft tissue vs. osteomyelitis) [36].

Polypharmacy leading to frequent drug–drug interactions are common among people with diabetes. Mallarino-Haeger et al. performed a retrospective study that included patients that were hospitalized with DFO and found that the median (IQR) number of medications prescribed on hospital discharge was 8 (6–11) [37]. Due to frequent comorbid conditions, medications other than anti-diabetic drugs are often prescribed to people living with diabetes. In a prospective observational study among inpatients with diabetes, Sankar et al. reported that the most common drugs associated with drug–drug interactions were cardiac drugs (92%) and non-steroid anti-inflammatory drugs (66%), followed by antibiotics (52%) [38]. Of note, none of the seven serious interactions identified in the screened prescriptions involved antibiotics.

Examples of drug–drug interactions between commonly used antibiotics and medications frequently prescribed to people with diabetes include by-mouth antiacids and iron preparations, which decrease the bioavailability of certain antibiotics, especially FQ and tetracyclines [38]. Rifampin is notorious for its many drug–drug interactions, including with anticoagulants and clopidogrel, an antiplatelet agent frequently prescribed to people with peripheral artery disease [35]. Fluoroquinolones, especially moxifloxacin, are QTc-prolonging, and clinicians should beware of co-prescribing multiple QTc-prolonging meds, particularly for older patients with heart disease. The co-administration of co-trimoxazole with angiotensin converting enzyme inhibitors or angiotensin receptor blockers may increase the risk of sudden death, particularly among older persons, likely due to hyperkalemia [39]. Intramuscular administration is usually contraindicated to people with DFO who frequently receive anticoagulants, although high-quality evidence supporting this precaution is sparse, and subcutaneous administration is not easy for most of the antibiotics used in this setting. Overall, data about potential effects of anti-diabetic agents on the oral absorption of antibiotics taken orally are lacking.

Peripheral artery disease and microcirculatory impairment, which are frequently associated with diabetes, are both suspected to impact the antibacterial activity and tissue availability of antibiotics in the foot. It is generally thought that high doses made possible by the intravenous administration of antibiotics can overcome these limitations [40]. Clinical studies published so far provide conflicting data on this issue. In a clinical study that compared two parenteral-to-oral antibiotic regimens, similar results were observed in patients with and without (noncritical) foot ischemia [41]. Raymakers et al. reported the minimal impact of tissue assessed by perfusion (TcPO2) on the availability of ceftazidime administered intravenously into different tissues, including bones [42]. A potential limitation of this study is that these conclusions were based on tissue concentrations assessed once during amputation procedures after a single dose of intravenous ceftazidime. In a more recent study, using a microdialysis catheter technique, Fejfarová et al. found that tissue concentration ≥ 50% fT > MIC of amoxicillin, and tissue concentration ≥ 60% fT > MIC of ceftazidime (both administered intravenously and assessed at steady state), were found in, respectively, 67% and 55% of the cases [43]. A significant positive correlation was established between maximum antibiotic tissue levels and their area under curve (AUC) with arterial flow but not with ankle/brachial index, toe/brachial index, or TcPO2 [43]. These results suggest a negative impact of peripheral artery disease on the bone diffusion of antibiotics in DFO, noting, however, that the molecules assessed were only beta-lactam agents given intravenously.

While achieving bone tissue at least at the minimal inhibitory concentration for causative microorganisms is necessary, it might not be sufficient in the case of chronic osteomyelitis because of the frequent involvement of biofilm. The antibiotic activity against biofilm-associated bacterial cells in reduced even quiescent status and embedded in a self-produced polymeric structure may be of interest in these settings unless all necrotic infected bone tissues have been removed during conservative surgery or amputation [44,45]. Only a very few studies have assessed the histopathology of bone samples in people with DFO. These studies provided conflicting results about the prevalence of chronic osteomyelitis probably in relation to the absence of the consensual histopathological definition of a “chronic DFO” and differences in the patients included especially regarding the earliness of bone surgery [46,47]. Baudoux et al. found that in DFOs, about two-thirds of bone samples contained biofilm assessed by both crystal violet staining and electron microscopy [48]. More recently, Johani et al. using the scanning electron microscopy technique confirmed these results with the identification of biofilm structures in 80% of the bone specimens [49]. Systemic antibiotics currently used are predominantly effective against planktonic microbial cells, with commonly used agents (such as beta-lactams and glycopeptides) only being effective in multiplying bacteria [50]. These results are consistent with the study from Tardáguila-García et al. [46] and provide arguments for the role of chronic osteomyelitis in people treated for DFO and potentially for using antibiotics with anti-biofilm activity in DFO episodes treated non-surgically (i.e., without any bone resection) [51].

Overall, several factors related to DFO-related features may negatively impact the oral administration of antibiotics and their diffusion into chronically infected bones.

### 1.3. Clinical Data on Oral Antibiotic Therapy for DFO

Among the studies that included patients with DFO treated with various oral antibiotic regimens, only a few of them compared the outcomes of oral versus parenteral regimens. In a randomized, multicenter, open-label trial of linezolid versus ampicillin-sulbactam/amoxicillin-clavulanate, Lipsky et al. enrolled 371 patients with DFIs, including 77 (20.7%) DFO episodes [41]. Similar outcomes were reported in inpatients and outpatients with initial intravenous therapy versus oral therapy (i.e., for linezolid, 77% vs. 83%, respectively; for amino-penicillin/beta-lactamase inhibitors, 68% vs. 72%, respectively). Three recent studies conducted by the same group from Switzerland enrolled large cohorts of patients with DFIs including DFO episodes treated in part with oral antibiotics [52,53,54]. The first one is a retrospective cohort study that found no influence of the route and duration of the antibiotic therapy on the risk of infection recurrence [52]. Among the 392 DFO episodes, 293 (74.7%) were treated with amputations at different levels and some others with bone debridement [52]. The second is a retrospective study that found similar outcomes of DFO episodes treated with more than 1 week of intravenous antibiotics compared with patients receiving less than 1 week of treatment [53]. The third study (RCT) compared two durations of oral antibiotic regiments with only a few days of initial intravenous administration in patients who all underwent surgical bone debridement for DFO, including partial amputations in 36% of cases [54]. Two more recent retrospective studies aimed to compare oral and intravenous antibiotic therapy for DFO. One reported by Kipp et al. found that complete wound healing and residual infections requiring further debridement or amputation in 128 patients treated with either oral or intravenous antibiotics for DFO were comparable [55]. Gill et al. reported similar outcomes of 65 patients who received either intravenous or oral antibiotic [56]. The results established in these two studies suggest that targeted oral antibiotics can be as safe and effective as intravenous antibiotics in selected patients, noting that all patients included in both studies had undergone foot amputation.

DFO is usually associated with concomitant skin and soft-tissue infections (SSTIs), which makes it difficult to distinguish, in the published studies, the antibiotic treatment of STTIs from that of DFO when both infections coexist and therefore to deteine precisely the duration and mode of administration of the antibiotics targeting DFO. The review of the few studies that provide data on the mode of administration of the antibiotic therapy shows that what is called “oral antibiotic therapy” is based on initial intravenous antibiotics for variable durations followed by oral antibiotics. This is a key knowledge gap that currently precludes guidelines from recommending a fully oral antibiotic course without an IV lead-in. Notably, the part of the intravenous administration of antibiotics ranges from less than 10% to more than 50% of the total of the antibiotic treatment (Table 2). In addition, the studies that assessed oral antibiotic regimens for DFO included patients who had undergone, for a significant proportion of them, bone resection, or minor or major amputation, which may also explain the differences in clinical remission rates from one study to another (Table 2). The available data are unlikely to apply to patients treated non-surgically for DFO as the role of the antibiotic therapy is obviously less important than in DFO treated with a medical (non-surgical) approach [44]. In total, none of these papers provide high-quality evidence to support oral-only antibiotic therapy for DFO treated non-surgically.

### 1.4. Is Oral-Only Antibiotic Therapy a Reasonable Option for DFO?

Clinical studies have shown that DFO could be arrested by using highly bioavailable oral agents even with a non-surgical approach [28,57,58,59]. For multiples reasons, dominated by the common beliefs that the intravenous administration of antibiotics is intrinsically more efficient than the oral route to treat bone infections, most patients with DFO continue to receive initial intravenous antibiotics for durations that vary from several days to several weeks (Table 2). The International Working Group on the Diabetic Foot/Infectious Diseases Society of America (IWGDF/IDSA) 2023 guidelines recommend parenteral antibiotics in cases of systemic signs of infection (i.e., severe or grade 4 infection), bacteraemia, and gangrene, which can be associated with DFO [61]. The intravenous antibiotic therapy recommended in these situations is related to the severity of the skin and soft tissue infections rather than to the bone infection. Switching to oral therapy is recommended if the patient is clinically improving, has no contraindications to oral therapy, and if there is an appropriate oral agent available [61]. Due to the knowledge gaps discussed above, no recommendations have been formulated about the possibility of treating DFO with oral-only antibiotic therapy. Advantages and disadvantages of full intravenous, intravenous, and then oral and oral-only antibiotic therapy are shown in Table 3.

Prerequisites for antibiotics administered orally depend on the therapeutical approach (e.g., surgical versus non-surgical) of DFO. The current literature suggests that any class of antibiotics, even those with low BPR and low oral bioavailability such as beta-lactam agents and glycopeptides, can be used in people with good results provided they have undergone bone debridement or amputation [52]. Remission of bone infection in non-surgical DFOs have mainly been obtained with antibiotics exhibiting high oral bioavailability and bone/plasma ratio such as FQ (e.g., ciprofloxacin, levofloxacin), clindamycin, oxazolidinones (e.g., linezolid and tedizolid), rifampicin, trimethoprim-sulfamethoxazole, or tetracyclines (e.g., doxycycline and minocycline). The available evidence on oral treatment for DFO may lead to the following proposals regarding the route of the antibiotic therapy:

(i) Antibiotics for DFO without associated SSTI are started orally and administered for the entire duration of treatment; these cases generally correspond to DFO episodes treated non-surgically.
(ii) Antibiotics are started intravenously for severe SSTI concomitant to DFO and are switched to oral route for the rest of the treatment as soon as the SSTI has improved (if antibiotics targeting DFO differ from those used for the SSTI, they are started orally).
(iii) Oral antibiotics are used for the entire duration of the treatment in non-severe SSTIs associated with DFO.

It must be noted, however, that the current increase of multidrug-resistant bacteria, especially in gram-negative rods, may progressively augment the need for intravenous-only broad-spectrum antibiotic therapies for which the oral route is not possible, such as carbapenems, cefiderocol, newer beta-lactam/beta-lactamase inhibitor combinations, aminoglycoside, or colistin. Importantly, each DFO episode requires an individualized approach because of the numerous variables that may affect the final decision, which ideally should be taken in multidisciplinary teams including senior physicians with skilled experience in this field.

In total, there are two ways to approach the issue of the oral treatment of DFO. One is to consider that there is enough of a rationale to recommend the use of oral antibiotics for DFO. Wiki guidelines have already been engaged in this path [64]. Another is to be more cautious, considering the gaps between the convincing studies in chronic osteomyelitis in general and the specific characteristics of DFO. This is the current position adopted by the IWGDF/IDSA guidelines. Of note, the accumulation of data in favor of oral versus intravenous antibiotics for the treatment of chronic osteomyelitis may limit, in the future, the acceptability of an RCT, with the aim of comparing oral-only antibiotics to full-intravenous or initial intravenous followed by oral antibiotics in patients with DFO.

## 2. Conclusions

Despite a growing body of evidence about the benefits of oral versus intravenous antibiotic therapy for people suffering from DFO on health outcomes, patients’ quality of life, and efficiency, intravenous antibiotics are still largely used, especially at the start of treatment. Well-designed clinical studies with the aim of confirming the cost/benefit value of oral versus intravenous antibiotics for DFO are still needed to change the medical consensus. Moreover, since well-powered clinical trials comparing different oral antibiotics for DFO are unlikely to be conducted [65], ongoing well-designed observational studies are needed to determine the associations between different oral antibiotics and treatment outcomes.

## Figures and Tables

**Table 1:** Antibiotic oral bioavailability (from ref. [24]).

Antibiotics	Oral Bioavailability (%)
Clindamycin, Doxycycline, Levofloxacin, Linezolid, Metronidazole, and Rifampicin	≈90
Amoxicillin *, **, Cephalexin *, Cipro/Moxifloxacin, and Trimethoprim-Sulfamethoxazole	>80 ≤ 90
Penicillin V *	>60 ≤ 80
Amoxicillin-clavulanic acid *, Cefixime/Cefpodoxime *, and Oxa */Cloxa */Dicloxacillin *	≤60

Note: all the antibiotics in the table have a bone/plasma concentration ratio ≥ 0.3, except beta-lactams agents (*), for which this ratio is >0.1 < 0.3 refs. [19,20,24]. **: oral biovailability depends on the dose administered: from 100% for 375 mg to 55% for 3000 mg.

**Table 2:** Studies that included patients with diabetes-related osteomyelitis of the foot treated with oral antibiotics.

Reference Year of Publication [Ref.]	Study Design	Bone Surgery	N° of DFO Episodes	Duration of the Antibiotic Therapy			Remission Rate (%)
				Intravenous	Oral	Total	
Lipsky 1997 [57]	RCT (C) Ofloxacin vs. BL/BLI	71%	21 ofloxacin BL/BIL	7.8 days, m 7.1 days, m	13.2 days, m 12.0 days, m	≈3 weeks, m	85 83
Senneville 2001 [58]	Retrospective cohort study	Necrotic bone resection in 2 cases (11.7%)	17	In 5 cases (29%): 5.5 days, M (range 3–17)	NR	6 months, M (range 3–10)	76
Lipsky 2004 [41]	RCT (C) Linezolid vs. ampicillin-sulbactam	*	77	7.8 ± 5.5 days, m 10.4 ± 5.7 days, m	15.9 ± 7.4 days, m 15.0 ± 7.8 days, m	17 days, m 17.2 ± 7.9 days, m 16.5 ± 7.9d days, m	61 69
Embil 2006 [59]	Retrospective cohort study	Bone debridement in 26 cases (28%), toe amputation in 9 cases (10%)	93	2 weeks, m	40 weeks, m	≈ 42 weeks	82
Senneville 2008 [60]	Retrospective cohort study	No bone surgery	50	In 16 cases (32%): one week	NR	11.5 ± 4.21 weeks, m	
Tone 2015 [28]	RCT (6 vs. 12 weeks)	No bone surgery	40	In 18 cases (45%): 8 days, M [IQR: 6–9]	NR	6 weeks, m (*n* = 20) 12 weeks, m (*n* = 20)	
Gariani 2019 [52]	Retrospective cohort study	Amputations (74.7%) or bone debridement	392	4 days, M (all patients received initial intravenous antibiotics)	NR	31–34 days, M	75
Gariani 2019 [53]	Retrospective cohort study	All patients	339	6 days, M (all patients with initial intravenous antibiotics)	22 days (M; 12–30)	NR	78
Gariani 2021 [54]	RCT (3 vs. 6 weeks)	**	93	1 day, M (*n* = 44) 3 days, M (*n* = 49)	20 days, M (*n* = 44) 39 days, M (*n* = 49)	3 weeks ± 2 days (*n* = 44) or 6 weeks ± 2 days (*n* = 49)	84 73
Kipp 2024 [55]	Retrospective cohort study (C)	Amputation in all patients	128 (oral = 54, IV = 74)	≥4 weeks	≥4 weeks	NR	66.7 (oral) 43.2 (IV)
Gill 2024 [56]	Retrospective cohort study (C)	Amputation in all patients	65 (oral = 35, IV = 30)	remission (*n* = 13): 5.8 ± 0.6 days, m failure (*n* = 17): 6.2 ± 1.5 days, m	remission (*n* = 20): 5.6 ± 1.3 days, m failure (*n* = 15): 5.7 ± 0.7 days, m	5.8 ± 1.1 days	50.8

Legend: (C): comparison of oral versus intravenous antibiotic therapy; RCT: randomized controlled study; IV = intravenous; and BL/BLI: Beta-lactam/Beta-lactamase inhibitor; NR = not reported. *: patients could have undergone any necessary debridement or other surgical procedures, if the entire infected area was not resected or amputation. **: median number of surgical debridement per episode was 1 (IQR, 0–2 interventions), among which 34 (36%) were partial amputations. m: mean ± standard deviation; M: median; and IQR: interquartile range.

**Table 3:** Comparative aspects of full-intravenous, 7-day intravenous then oral, and oral-only administration of high oral-availability antibiotics (according to data from refs. [13,62,63]).

Route of Administration	Bioavailability	Adherence (If the Patient Remains Hospitalized While on IV Antibiotics)	Catheter-Related Complications	Length of Hospitalization Including OPAT	Costs	Efficacy
Full-intravenous	complete	complete	9.4–21%	14 days [IQR: 11–21]	+++	similar
7-day intravenous, then oral	complete for 7 days then oral-antibiotic dependent	complete for 7 days, then variable	1%	11 days [IQR: 8–20]	++	
Full-oral	oral-antibiotic dependent	variable	0%	no data	+	no data (particularly in the absence of surgery)

Legend: OPAT: outpatient antibiotic therapy; IQR: interquartile range.

## Data Availability

No underlying data are available for this article, since no datasets were generated or analyzed during this study.

## References

[1] Xing K., Huang G., Hua S., Xu G., Li M. (2019). Systematic review of randomized controlled trials on antibiotic treatment for osteomyelitis in diabetes. Diab. Med..

[2] Cortes-Penfield N.W., Armstrong D.G., Brennan M.B., Fayfman M., Ryder J.H., Tan T.W., Schechter M.C. (2023). Evaluation and Management of Diabetes-related Foot Infections. Clin. Infect. Dis..

[3] Armstrong D.G., Tan T.W., Boulton A.J.M., Bus S.A. (2023). Diabetic Foot Ulcers A Review. JAMA.

[4] Jhaveri V.V., Sullivan C., Ward A., Giurini J.M., Karchmer A.W., Stillman I.E., Davis R.B., Freed J.A., LaSalvia M.T., Stead W. (2022). More Specialties, Fewer Problems: Using Collaborative Competency Between Infectious Diseases, Podiatry, and Pathology to Improve the Care of Patients with Diabetic Foot Osteomyelitis. J. Am. Podiatr. Med. Assoc..

[5] Peters E.J.G., Albalawi Z., van Asten S.A., Abbas Z.G., Allison G., Aragón-Sánchez J., Embil J.M., Lavery L.A., Alhasan M., Oz O. (2024). Interventions in the management of diabetes-related foot infections: A systematic review. Diabetes Metab. Res. Rev..

[6] Greenberg R.N., Tice A.D., Marsh P.K., Craven P.C., Reilly P.M., Bollinger M., Weinandt W.J. (1987). Randomized trial of ciprofloxacin compared with other antimicrobial therapy in the treatment of osteomyelitis. Am. J. Med..

[7] Gentry L.O., Rodriguez G.G. (1990). Oral ciprofloxacin compared with parenteral antibiotics in the treatment of osteomyelitis. Antimicrob. Agents Chemother..

[8] Mader J.T., Cantrell J.S., Calhoun J. (1990). Oral ciprofloxacin compared with standard parenteral antibiotic therapy for chronic osteomyelitis in adults. J. Bone Jt. Surg. Am..

[9] Gentry L.O., Rodriguez-Gomez G. (1991). Ofloxacin versus parenteral therapy for chronic osteomyelitis. Antimicrob. Agents Chemother..

[10] Gomis M., Barberan J., Sanchez B., Khorrami S., Borja J., Garcia-Barbal J. (1999). Oral ofloxacin versus parenteral imipenem-cilastatin in the treatment of osteomyelitis. Rev. Esp. Quimioter..

[11] Schrenzel J., Harbarth S., Schockmel G., Genné D., Bregenzer T., Flueckiger U., Petignat C., Jacobs F., Francioli P., Zimmerli W. (2004). A randomized clinical trial to compare fleroxacin-rifampicin with flucloxacillin or vancomycin for the treatment of staphylococcal infection. Clin. Infect. Dis..

[12] Euba G., Murillo O., Fernández-Sabé N., Mascaró J., Cabo J., Pérez A., Tubau F., Verdaguer R., Gudiol F., Ariza J. (2009). Long-term follow-up trial of oral rifampin-cotrimoxazole combination versus intravenous cloxacillin in treatment of chronic staphylococcal osteomyelitis. Antimicrob. Agents Chemother..

[13] Li H.K., Rombach I., Zambellas R., Walker A.S., McNally M.A., Atkins B.L., Lipsky B.A., Hughes H.C., Bose D., Kümin M. (2019). OVIVA Trial Collaborators. Oral versus intravenous antibiotics for bone and joint infection. N. Eng. J. Med..

[14] Manning L., Metcalf S., Dymock M., Robinson O., Clark B., Nelson R., Paterson D.L., Yates P., Loewenthal M., Dewar D. (2022). Short- versus standard-course intravenous antibiotics for peri-prosthetic joint infections managed with debridement and implant retention: A randomised pilot trial using a desirability of outcome ranking (DOOR) endpoint. Int. J. Antimicrob. Agents.

[15] Obremskey W.T., O’Toole R.V., Morshed S., Tornetta P., Murray C.K., Jones C.B., Scharfstein D.O., Taylor T.J., Carlini A.R., Major Extremity Trauma Research Consortium (METRC) (2025). Oral vs Intravenous Antibiotics for Fracture-Related Infections: The POvIV Randomized Clinical Trial. JAMA Surg..

[16] Wald-Dickler N., Holtom P.D., Phillips M.C., Centor R.M., Lee Rachael A., Baden R., Spellberg B. (2022). Oral Is the New IV. Challenging Decades of Blood and Bone Infection Dogma: A Systematic Review. Am. J. Med..

[17] Hawkins M.R., Thottacherry E., Juthani P., Aronson J., Chang A., Amanatullah D.F., Markovits J., Shen S., Holubar M., Andrews J.R. (2024). Implementing Oral Antibiotics for Bone and Joint Infections: Lessons Learned and Opportunities for Improvement. Open Forum Infect. Dis..

[18] Phillips M.C., Wald-Dickler N., Davar K., Lee R., Baden R., Holtom P., Spellberg B. (2023). Choosing patients over placebos: Oral transitional therapy vs. IV-only therapy for bacteraemia and infective endocarditis. Clin. Microbiol. Infect..

[19] Landersdorfer C.B., Bulitta J.B., Kinzig M., Holzgrabe U., Sorgel F. (2009). Penetration of antibacterials into bone: Pharmacokinetic, pharmacodynamic and bioanalytical considerations. Clin. Pharmacokinet..

[20] Thabit A.K., Fatani D.F., Bamakhrama M.S., Barnawi O.A., Basudan L.O., Alhejaili S.F. (2019). Antibiotic penetration into bone and joints: An updated review. Int. J. Infect. Dis..

[21] Traunmüller F., Schintler M.V., Spendel S., Popovic M., Mauric O., Scharnagl E., Joukhadar C. (2010). Linezolid concentrations in infected soft tissue and bone following repetitive doses in diabetic patients with bacterial foot infections. Int. J. Antimicrob. Agents.

[22] Schintler M.V., Traunmüller F., Metzler J., Kreuzwirt G., Spendel S., Mauric O., Popovic M., Scharnagl E., Joukhadar C. (2009). High fosfomycin concentrations in bone and peripheral soft tissue in diabetic patients presenting with bacterial foot infection. J. Antimicrob. Chemother..

[23] Traunmüller F., Schintler M.V., Metzler J., Spendel S., Mauric O., Popovic M., Konz K.H., Scharnagl E., Joukhadar C. (2010). Soft tissue and bone penetration abilities of daptomycin in diabetic patients with bacterial foot infections. J. Antimicrob. Chemother..

[24] Eleftheriotis G., Marangos M., Lagadinou M., Bhagani S., Assimakopoulos S.F. (2023). Oral Antibiotics for Bacteremia and Infective Endocarditis: Current Evidence and Future Perspectives. Microorganisms.

[25] Spellberg B., Lipsky B.A. (2012). Systemic antibiotic therapy for chronic osteomyelitis in adults. Clin. Infect. Dis..

[26] Ball C.S., Manson J.M., Reid F., Tweedle D.E. (1989). The pharmacokinetics of the biliary excretion of ciprofloxacin. HPB Surg..

[27] Holmes N.E., Charles P.G.P. (2009). Safety and Efficacy Review of Doxycycline. Clin. Med. Ther..

[28] Tone A., Nguyen S., Devemy F., Topolinski H., Valette M., Cazaubiel M., Fayard A., Beltrand É., Lemaire C., Senneville É. (2015). Six-week versus twelve-week antibiotic therapy for nonsurgically treated diabetic foot osteomyelitis: A multicenter open-label controlled randomized study. Diab. Care.

[29] Lesens O., Desbiez F., Theïs C., Ferry T., Bensalem M., Laurichesse H., Tauveron I., Beytout J., Aragón Sánchez J., Working Group on Diabetic Osteomyelitis (2015). Staphylococcus aureus-Related Diabetic Osteomyelitis: Medical or Surgical Management? A French and Spanish Retrospective Cohort. Int. J. Low. Extrem. Wounds.

[30] van Asten S.A.V., Mithani M., Peters E.J.G., La Fontaine J., Kim P.J., Lavery L.A. (2018). Complications during the treatment of diabetic foot osteomyelitis. Diab. Res. Clin. Pract..

[31] Metwally A.S., El-Sheikh S.M.A., Galal A.A.A. (2022). The impact of diabetes mellitus on the pharmacokinetics of rifampicin among tuberculosis patients: A systematic review and meta-analysis study. Diab. Metab Syndr..

[32] Zhu Y., Forsman L.D., Chen C., Zhang H., Shao G., Wang S., Wang S., Xiong H., Bruchfeld J., Wang W. (2024). Drug Exposure and Treatment Outcomes in Patients With Multidrug-Resistant Tuberculosis and Diabetes Mellitus: A Multicenter Prospective Cohort Study From China. Clin. Inf. Dis..

[33] Adithan C., Sriram G., Swaminathan R.P., Shashindran C.H., Bapna J.S., Krishnan M., Chandrasekar S. (1989). Differential effect of type I and type II diabetes mellitus on serum ampicillin levels. Int. J. Clin. Pharmacol. Ther. Toxicol..

[34] Marangos M.N., Skoutelis A.T., Nightingale C.H., Zhu Z., Psyrogiannis A.G., Nicolau D.P., Bassaris H.P., Quintiliani R. (1995). Absorption of ciprofloxacin in patients with diabetic gastroparesis. Antimicrob. Agents Chemother..

[35] Cevik M., Sturdy A., Maraolo A.E., Dekkers B.G.J., Akkerman O.W., Gillespie S.H., Alffenaar J.W.C. (2024). A systematic review on the effect of diabetes mellitus on the pharmacokinetics of TB drugs. Int. J. Tuberc. Lung Dis..

[36] Sanz-Corbalán I., Tardáguila-García A., García-Álvarez Y., López-Moral M., Álvaro-Afonso F.J., Lázaro-Martínez J.L. (2024). Evaluation of Adherence to the Oral Antibiotic Treatment in Patients With Diabetic Foot Infection. Int. J. Low. Extrem. Wounds.

[37] Mallarino-Haeger C., Watson A., Mahgoub U., Francis L., Heydari M., Choudhary M., Garcia-Toca M., Patel M., Kempker R.R., Fayfman M. (2024). High Prescription Rate of Medications With Rifampin Drug-drug Interactions in Patients With Diabetic Foot Osteomyelitis: Should Rifabutin Be Included in Clinical Trials for Adjunctive Therapy?. Open Forum Inf. Dis..

[38] Sankar V., Saaed Y., Joseph R.M., Azizi H., Thomas P.M. (2015). Serious Drug-Drug Interactions in the Prescriptions of Diabetic Patients. Med. Sci..

[39] Fralick M., Macdonald E.M., Gomes T., Antoniou T., Hollands S., Mamdani M.M., Juurlink D.N., Canadian Drug Safety and Effectiveness Research Network (2014). Co-trimoxazole and sudden death in patients receiving inhibitors of renin-angiotensin system: Population based study. BMJ.

[40] McCarthy K., Avent M. (2020). Oral or intravenous antibiotics?. Aust. Prescr..

[41] Lipsky B.A., Itani K., Norden C. (2004). Linezolid Diabetic Foot Infections Study Group. Treating foot infections in diabetic patients: A randomized, multicenter, open-label trial of linezolid versus ampicillin-sulbactam/amoxicillin-clavulanate. Clin. Infect. Dis..

[42] Raymakers J.T., Houben A.J., van der Heyden J.J., Tordoir J.H., Kitslaar P.J., Schaper N.C. (2001). The effect of diabetes and severe ischaemia on the penetration of ceftazidime into tissues of the limb. Diabet. Med..

[43] Fejfarová V., Jarošíková R., Antalová S., Husáková J., Wosková V., Beca P., Mrázek J., Tůma P., Polák J., Dubský M. (2024). Does PAD and microcirculation status impact the tissue availability of intravenously administered antibiotics in patients with infected diabetic foot? Results of the DFIATIM substudy. Front. Endocrinol..

[44] Senneville E., Robineau O. (2017). Treatment options for diabetic foot osteomyelitis. Expert Opin. Pharmacother..

[45] Zelmer A.R., Yang D., Gunn N.J., Solomon L.B., Nelson R., Kidd S.P., Richter K., Atkins G. (2024). JOsteomyelitis-relevant antibiotics at clinical concentrations show limited effectivity against acute and chronic intracellular *S. aureus* infections in osteocytes. Antimicrob. Agents Chemother..

[46] Tardáguila-García A., Sanz-Corbalán I., García-Morales E., García-Álvarez Y., Molines-Barroso R.J., Lázaro-Martínez J.L. (2021). Diagnostic Accuracy of Bone Culture Versus Biopsy in Diabetic Foot Osteomyelitis. Adv. Ski. Wound Care.

[47] Aragón-Sánchez F.J., Cabrera-Galván J.J., Quintana-Marrero Y., Hernández-Herrero M.J., Lázaro-Martínez J.L., García-Morales E., Beneit-Montesinos J.V., Armstrong D.G. (2008). Outcomes of surgical treatment of diabetic foot osteomyelitis: A series of 185 patients with histopathological confirmation of bone involvement. Diabetologia.

[48] Baudoux F., Neut C., Beltrand E., Lancelevée J., Lebrun C., Lemoux O., Vambergue A., Dubreuilh L., Fontaine P., Senneville E. (2012). Facteurs de pathogénicité dans l’ostéite du pied diabétique: Étude de la charge bactérienne et de la capacité à former un biofilm. Diabetes Metab..

[49] Johani K., Fritz B.G., Bjarnsholt T., Lipsky B.A., Jensen S.O., Yang M., Dean A., Hu H., Vickery K., Malone M. (2019). Understanding the microbiome of diabetic foot osteomyelitis: Insights from molecular and microscopic approaches. Clin. Microbiol. Infect..

[50] Lavigne J.P., Sotto A. (2017). Microbial management of diabetic foot osteomyelitis. Future Microbiol..

[51] Senneville E., Gachet B., Blondiaux N., Robineau O. (2023). Do Anti-Biofilm Antibiotics Have a Place in the Treatment of Diabetic Foot Osteomyelitis?. Antibiotics.

[52] Gariani K., Lebowitz D., von Dach E., Kressmann B., Lipsky B.A., Uçkay I. (2019). Remission in diabetic foot infections: Duration of antibiotic therapy and other possible associated factors. Diabetes Obes. Metab..

[53] Gariani K., Lebowitz D., Kressmann B., von Dach E., Sendi P., Waibel F., Berli M., Huber T., Lipsky B.A., Uçkay I. (2018). Oral amoxicillin-clavulanate for treating diabetic foot infections. Diabetes Obes. Metab..

[54] Gariani K., Pham T.T., Kressmann B., Jornayvaz F.R., Gastaldi G., Stafylakis D., Philippe J., Lipsky B.A., Uçkay L. (2021). Three Weeks Versus Six Weeks of Antibiotic Therapy for Diabetic Foot Osteomyelitis: A Prospective, Randomized, Noninferiority Pilot Trial. Clin. Infect. Dis..

[55] Kipp J.A., LeSavage L.K., Evans J.K., Denmeade T.A., Blazek C.D. (2024). Diabetic Osteomyelitis: Oral versus Intravenous Antibiotics at a Single Level 1 Academic Medical Trauma Center. J. Foot Ankle Surg..

[56] Gill A.S., Gorski M., Strage K.E., Dunn J.T., Jerabek M., Hoffman K.M. (2022). Oral Versus Intravenous Antibiotics for Residual Osteomyelitis After Amputation in the Diabetic Foot. J. Foot Ankle Surg..

[57] Lipsky B.A., Baker P.D., Landon G.C., Fernau R. (1997). Antibiotic therapy for diabetic foot infections: Comparison of two parenteral-to-oral regimens. Clin. Infect. Dis..

[58] Senneville E., Yazdanpanah Y., Cazaubiel M., Cordonnier M., Valette M., Beltrand E., Khazarjian A., Maulin L., Alfandari S., Caillaux M. (2001). Rifampicin-ofloxacin oral regimen for the treatment of mild to moderate diabetic foot osteomyelitis. J. Antimicrob. Chemother..

[59] Embil J.M., Rose G., Trepman E., Math M.C.M., Duerksen F., Simonsen J.N., Nicolle L.E. (2006). Oral Antimicrobial Therapy for Diabetic Foot Osteomyelitis. Foot Ankle Int..

[60] Senneville E., Lombart A., Beltrand E., Valette M., Legout L., Cazaubiel M., Yazdanpanah Y., Fontaine P. (2008). Outcome of diabetic foot osteomyelitis treated non-surgically: A retrospective cohort study. Diabetes Care.

[61] Senneville É., Albalawi Z., van Asten S.A., Abbas Z.G., Allison G., Aragón-Sánchez J., Embil J.M., Lavery L.A., Alhasan M., Oz O. (2024). IWGDF/IDSA guidelines on the diagnosis and treatment of diabetes-related foot infections (IWGDF/IDSA 2023). Diabetes Metab. Res. Rev..

[62] Pulcini C., Couadau T., Bernard E., Lorthat-Jacob A., Bauer T., Cua E., Mondain V., Chichmanian R.M., Dellamonica P., Roger P.M. (2008). Adverse effects of parenteral antimicrobial therapy for chronic bone infections. Eur. J. Clin. Microbiol. Infect. Dis..

[63] Hoffman-Terry M.L., Fraimow H.S., Fox T.R., Swift B.G., Wolf J.E. (1999). Adverse effects of outpatient parenteral antibiotic therapy. Am. J. Med..

[64] Spellberg B., Aggrey G., Brennan M.B., Footer B., Forrest G., Hamilton F., Minejima E., Moore J., Ahn J., Angarone M. (2022). Use of Novel Strategies to Develop Guidelines for Management of Pyogenic Osteomyelitis in Adults: A WikiGuidelines Group Consensus Statement. JAMA Netw. Open.

[65] Schechter M.C., Sax P.E., Cortés-Penfield N. (2022). What Is the Best Oral Therapy for *Staph aureus* Osteomyelitis?. NEJM Evid..

